# Supplementary material for: Direct Transport to Cardiac Arrest Center and Survival Outcomes after Out-of-Hospital Cardiac Arrest by Urbanization Level
Source: J Clin Med. 2022 Feb 16;11(4):1033. doi: 10.3390/jcm11041033 (PMC8877090; doi:10.3390/jcm11041033)
Supplement: Supplementary file 1 [file jcm-11-01033-s001.zip › jcm-1582476-supplementary.pdf]

Supplementary

Table S1. Sensitivity analysis of multivariable logistic regression model for pulseless out-of-hospital cardiac arrest patients who did not achieve prehospital ROSC.

	Total	Outcome		Model 1	Model 2	Model 3
	n	*n*	%	aOR (95% CI)	aOR (95% CI)	aOR (95% CI)
Good neurological recovery						
Transported hospital						
Non-cardiac center	65,299	242	0.4	1.00	1.00	1.00
Cardiac center	19,312	139	0.7	1.46 (1.17–1.82)	1.37 (1.09–1.72)	1.49 (1.18–1.87)
Urbanization level						
Urban/rural area	50,825	147	0.3	1.00	1.00	1.00
Metropolitan area	33,786	234	0.7	2.12 (1.71–2.64)	2.07 (1.66–2.58)	1.91 (1.52–2.40)
Survival to discharge						
Transported hospital						
Non-cardiac center	65,299	1,181	1.8	1.00	1.00	1.00
Cardiac center	19,312	606	3.1	1.45 (1.31–1.61)	1.36 (1.23–1.52)	1.45 (1.30–1.61)
Urbanization level						
Urban/rural area	50,825	810	1.6	1.00	1.00	1.00
Metropolitan area	33,786	977	2.9	1.64 (1.48–1.81)	1.59 (1.43–1.75)	1.46 (1.32–1.62)

aOR, adjusted odds ratio; CI, confidence interval.Model 1: adjusted for age and sex. Model 2: adjusted for variables in Model 1, comorbidities (diabetes mellitus, hypertension, and heart disease), place of arrest, witness status, bystander CPR, and initial shockable rhythm. Model 3: adjusted for variables in Model 2, response time interval, scene time interval, transport time interval, multi-tier response, EMS airway management, and mechanical CPR.
